# Predictors of adverse birth outcomes among pregnant adolescents in Ashanti Region, Ghana

**DOI:** 10.1017/jns.2021.58

**Published:** 2021-08-23

**Authors:** Reginald Adjetey Annan, Linda Afriyie Gyimah, Charles Apprey, Odeafo Asamoah-Boakye, Linda Nana Esi Aduku, Wisdom Azanu, Herman E. Luterodt, Anthony K. Edusei

**Affiliations:** 1 Human Nutrition and Dietetics Unit, Department of Biochemistry and Biotechnology, Faculty of Biosciences, College of Science, Kwame Nkrumah University of Science and Technology, Kumasi, Ghana; 2 Department of Obstetrics and Gynecology, University of Allied Health Sciences, Ho, Ghana; 3 Department of Food Science and Technology, Kwame Nkrumah University of Science and Technology, Kumasi, Ghana; 4 Department of Community Health, School of Public Health, Kwame Nkrumah University of Science and Technology, Kumasi, Ghana

**Keywords:** Adolescent pregnancy, Birth outcomes, Household hunger scale, Lived poverty index, Low birth weight, Preterm

## Abstract

Adolescent pregnancy is associated with adverse birth outcomes. However, the determinants of these outcomes are understudied. The present study sought to identify the predictors of adverse birth outcomes among pregnant adolescents in Ghana. In this prospective health centre-based study, 416 pregnant adolescents, aged 13–19 years old, were followed, and 270 birth outcomes were evaluated. We collected data on socio-demographic variables, eating behaviour, household hunger scale (HHS), lived poverty index (LPI) and compliance to antenatal interventions. The prevalence of low birth weight (LBW) and preterm births (PTB) were 15⋅2 and 12⋅5 %, respectively. Pregnant adolescents with no formal education (AOR 9⋅0; *P* = 0⋅004; 95 % CI 2⋅1, 39⋅8), those who experienced illness (AOR 3⋅0; *P* = 0⋅011; 95 % CI 1⋅3, 7⋅0), those who experienced hunger (OR 2⋅9; *P* = 0⋅010; 95 % CI 1⋅3, 6⋅5) and those with high LPI (OR 2⋅5; *P* = 0⋅014; 95 % CI 1⋅2, 5⋅3) presented increased odds of delivering preterm babies compared with those who have had secondary education, did not experience any illness, were not hungry or having low LPI, respectively. Pregnant adolescents who used insecticide-treated net (ITN) (AOR 0⋅4; *P* = 0⋅013; 95 % CI 0⋅2, 0⋅9) presented reduced odds LBW children; while those who experienced illness (AOR 2⋅7; *P* = 0⋅020; 95 % CI 1⋅2, 6⋅0), poorer pregnant adolescents (OR 2⋅5; *P* = 0⋅014; 95 % CI 1⋅1, 4⋅8) and those who experienced hunger (AOR 3⋅0; *P* = 0⋅028; 95 % CI 1⋅1, 8⋅1) presented increased odds of LBW children compared with those who used ITN, were not ill, were not poor or did not experience hunger. Adverse birth outcomes were associated with ANC compliance and socioeconomic factors of the pregnant adolescents. Hence, strengthening antenatal uptake and compliance by pregnant adolescents, promoting their livelihood and socioeconomic status, and interventions to prevent teenage pregnancies are strongly recommended.

## Introduction

Events that occur during the first 1000 d (from conception through to 2 years) in a child's life, including birth outcomes, are significant. They contribute to the commencement of proper development and impact future health^(1)^. Birth outcomes, including birth weight, gestational age, abortion, neonatal mortality and stillbirths, are a measure of babies’ health at birth. Of these, the two most studied indicators are birth weight and gestational age, yet their causes are not fully understood^(2)^. Worldwide, low birth weight (LBW) is estimated at 15–20 % births^(3)^, and 1⋅1 million babies die as a result of preterm birth complications annually^(4)^. The WHO estimates that more than 9 million infants were dying within their first birthday or even before birth every year, especially in developing countries^(5)^. The inequality in birth outcomes between low- and high-income countries is attributed to poor antenatal care (ANC) received by pregnant women in low-income countries^(6)^. Birth outcomes are generally related to social, economic, biological and environmental factors^(7–10)^.

Maternal nutrition practices, child growth period and the intrauterine environment during pregnancy may also affect birth outcomes^(11–14)^. A study conducted in Ghana on birth weight concluded that mothers with adequate dietary practices were less likely to have babies with LBW^(15)^. Poor maternal dietary habits have been related to rural living, food insecurity and poverty^(16)^. Moreover, unfavourable birth outcomes have been related to low socioeconomic status such as low education level, low income, poor access to health facilities and poor housing conditions^(17,18)^. Adverse birth outcomes have also been related to maternal morbidity and insufficient maternal care^(19–21)^, thus, early identification of complications of pregnancy during antenatal is vital.

ANC is the basic component of maternal care on which the life of mothers and babies depend^(22)^. According to the WHO^(23)^, ANC is part of the three most important care given to pregnant women and a crucial indicator of reducing the global maternal mortality ratio as specified in target 3 of the Sustainable Development Goal (SDG). Accessing ANC services have been directly linked to better birth outcomes and a decrease in infant mortality and malnutrition among low-income and middle-income countries^(24)^. ANC services are also related to socio-demographic or reproductive characteristics, and consequently birth outcomes^(25–28)^. The Government of Ghana endorsed the Focused Antenatal Care (FANC) strategy of the World Health Organization (WHO) in 2002 to address the significantly higher maternal mortality rate and to increase access, quality and consistency of ANC for pregnant women. ANC is a focal point for pregnant women and their fetuses for receiving a wide variety of preventive and healthcare activities, treatment, monitoring during pregnancy up to delivery^(29)^. There is late initiation of ANC attendance among pregnant adolescents as they are more likely to hide their pregnancy due to doubt and naivety, lack of finance and stigmatisation^(30)^. Studies among pregnant adolescents have reported a high risk for adverse birth outcomes, such as preterm, fetal death or LBW^(31–35)^. Furthermore, findings have reported an even higher risk for unfavourable birth outcomes in much younger adolescents^(36,37)^. The WHO^(38)^ reported an estimation of 12⋅8 million births (44 births per 1000 adolescent girls) of teenage girls between 15 and 19 years in 2018, with the highest occurrence in low-income countries (8 times higher), compared with high-income countries.

A maternal health survey conducted in 2017 in Ghana identified 12 % miscarried pregnancies, 2 % stillbirths and 10 % induced abortions among women aged 15–49 years^(39)^. Among these women of reproductive age, the prevalence of adolescent pregnancy was 14⋅2 %, with stillbirths, miscarriages and induced abortion recording 1⋅3, 6⋅8 and 18⋅8 %, respectively. Live births among this group were the least (73⋅1 %) as compared with the adult group (77⋅4 %), who were between 20 and 34 years^(39)^. A study conducted in Kumasi, Ghana, recorded a prevalence of 19 % adverse birth outcomes^(40)^ while an earlier one reported a 44⋅6 % prevalence^(41)^ among pregnant women. Among adolescents in Kumasi, the second-largest city in Ghana, 23 % had LBW^(42)^.

The seventh target of the SDG 3 seeks to ensure universal access to sexual and reproductive healthcare services. To achieve this goal, the Ministry of Gender, Children and Social Protection in Ghana developed a five-year strategic plan (2018–22). The plan seeks to reach adolescents with the correct information and insight and to provide them with training and services to protect them from early and unintended pregnancies^(43)^. Despite this, adolescent pregnancies seem to be on the rise. To prepare them for desirable birth outcomes, a better understanding of the determinants of birth outcomes in this group is required. WHO's global target for 2025 is to reduce LBW by 30 %, that is, a 3 % annually reduction. It is expedient that we identify the factors which lead to these adverse birth outcomes among this age bracket. The present study was, therefore, conducted to explore the predictors of adverse birth outcomes among pregnant adolescents in Ashanti Region, Ghana.

## Methods

### Study design

In this prospective health centre-based study, 416 pregnant adolescents (aged 13–19 years old) with a maximum of 32 gestational weeks were recruited from May to December 2018 and followed until they gave birth between January and July 2019.

### Study area and population

Ashanti Region, the most populated region in Ghana, makes up 19 % of Ghana's total population, which is about 5 792 187 people^(44)^. The prevalence of adolescent pregnancy in the region is 12⋅2 %, according to a recent survey conducted in 2017^(39)^. We recruited participants from health centres serving communities in three urban districts (Kumasi Metropolis, Asante Akim Central Municipal and Ejisu Juaben Municipal) and five rural districts (Bosomtwi, Asante Akim South and North, and Ahafo Ano North and South) in Ashanti Region, Ghana. We recurited participants at selected Community-based Health Planning Services (CHPS) compounds, health centres and hospitals in the selected districts^(44)^.

### Sample size

The sample size was statistically calculated using the sample size-based proportion formula by^(45)^: *n* =  2(*Z α*/2  +  *Z β* )2 *p*(1 − *p*)/(*P*1 − *P*2)^2^. Where *n* is the sample size, *Z α*/2 = 1⋅96 at type 1 error of 5 %, Z *β* = 0⋅84 at 80 % power, *P*1 is the LBW in pregnant adolescents with adequate nutritional status, *P*2 is the LBW in pregnant adolescents with poor nutritional status, *P*1 − *P*2 is the difference in the prevalence of LBW between pregnant adolescents with adequate nutritional status at birth and those with inadequate nutritional status, and *P* is pooled prevalence = (*P*1 + *P*2)/2. Based on a previous pilot study by Ayensu *et al.*^(42)^, which reported 23 % LBW prevalence, we proposed that LBW in pregnant adolescents with adequate nutritional status would be 11⋅4 %, while those with poor nutritional status would remain 23 %. Using the above descriptives, the sample size *n* = 2(1⋅96 + 0⋅84)^2^ × 0⋅1735(1–0⋅233)/(0⋅114–0⋅233)^2^, *n* = 2⋅09/0⋅01, *n* = 209 was calculated, which implied we needed to recruit 209 participants in each arm of the study (half in the poorly nourished group and a half in the well-nourished group) making 418 participants to show a significant association between poor nutrition and LBW. We added 10 % attrition to give 460 participants who were needed.

However, 416 pregnant adolescents were recruited on a first-come, first-served basis for the baseline study. Trained enumerators visited hospitals/health centres selected for the study on antenatal clinic days for pregnant adolescents. All pregnant adolescents within the inclusion criteria who gave written consents were written. In the rural areas, announcements were made at the community information centres to invite participants to the health centres on specific days. These announcements were necessary as some pregnant adolescents refused to visit the health centres for ANC to fear being stigmatised. We trained enumerators in a two-day workshop on each data collection tool used. We obtained birth outcome data from 270 (birth weight) and 303 (gestational age) participants out of the 416 recruited during the baseline study as some participants were lost to follow-up.

### Ethics

Ethical approval for the study was obtained from the Committee on Human Research Publication and Ethics (CHRPE) of the Kwame Nkrumah University of Science and Technology, KNUST, (Kumasi, Ghana) (CHPRE/AP/236/18). Study protocols/aims were first explained to all participants in their local language. Written and signed informed consent was obtained from all participants by following CHRPE regulations before recruiting for the study.

### Data collection tools

A structured questionnaire was used to collect data on the socio-demographic variables, dietary diversity, eating behaviours (pica, food aversion, food craving), food deprivation (household hunger scale – HHS), availability of necessities (lived poverty index – LPI), antenatal interventions compliance (nutrition education, micronutrient supplementation, tetanus injection, malaria tablet intake, use of the ITN, daily intake of micronutrient), maternal factors (maternal morbidity and mode of delivery) and birth outcomes (birth weight and gestational age) of the pregnant adolescents. Data on age and parity were verified from their National Health Insurance Identification cards and maternal health record book. The questionnaire was pretested at selected health centres and validated to ensure appropriate responses from participants. Birth weight and gestational age were the dependent variables.

### Assessment of dietary diversity

A previous day's 24-h dietary recall method was used to assess dietary diversity among the participants. The FAO's Minimum Dietary Diversity for Women (MDD-W), was used to determine maternal dietary diversity using ten food groups. The MDD-W score obtained was then used to identify whether maternal dietary diversity was adequate (5–10 food groups) or not (0–4 food groups).

### Assessment of eating behaviour

Questions on whether or not the participants were practising food cravings, pica and food aversions were asked. Each question had a yes/no optional response.

### Assessment of HHS and LPI

We collected data on food availability and deprivation over the past month using standardised close-ended questionnaires^(46)^ to assess hunger (HHS) among participants. The responses to these questions were then coded and scored. Scores between <2 meant little or no hunger, 2–3 meant moderate hunger and 4–6 meant severe hunger. LPI was assessed by asking questions on the availability of food, water, cash income, medical care and cooking fuel over the past year. The responses were coded and scored. A score of 0 meant no lived poverty, while the scores of 1–4 meant the absence of one or more necessities^(47)^. The averages of the scores were then categorised into low (0–1⋅0), moderate (1⋅01–1⋅5) and high (>1⋅5) LPI.

### Assessment of antenatal interventions

A structured questionnaire was administered to participants to identify their compliance with the antenatal interventions given at their various health facilities. Participants were asked if they received nutrition education, tetanus injection, micronutrient supplements, malaria tablet, ITN, consumed daily micronutrient supplement, and whether they used the ITN given.

### Assessment of birth outcomes and maternal factors

Follow-up data on several birth outcomes, including birth weight and gestational age at birth were obtained from their maternal health record books and were categorised as normal or adverse. LBW was defined as a birth weight of less than 2⋅5 kg^(28)^. Preterm birth was defined as delivery less than 37 completed weeks of gestation^(48)^. Questions on mode of delivery (vaginal delivery, caesarean section) and morbidity during pregnancy were asked.

### Data analysis

Microsoft Excel was used to clean the entered data, and Statistical Package for Social Sciences version 25 (SPSS IBM Inc., Chicago, USA) was used to analyse them. We performed *χ*^2^ (Fischer's exact test) and cross-tabulation to compare frequencies of all variables used (antenatal interventions and compliance, maternal factors and birth outcomes) among community types. Frequencies of birth weight and gestational age were compared among socio-demographic variables, dietary diversity, eating behaviours, LPI, HHS, antenatal interventions compliance and maternal factors using Fischer's exact test. We used logistic regression to identify predictors of birth outcomes. We presented the data as frequency (%) and *P* values < 0⋅05 were considered significant.

## Results

### Impact of socio-demographic factors on birth outcomes

The distribution of socio-demographic characteristics of pregnant adolescents and among community types used for the present study has been presented in another study^(16)^. Effects of socio-demographic status on birth outcomes are presented in Table 1. Among all the factors used, the level of education affected the gestational age at birth significantly (*P* = 0⋅006). Generally, preterm births (PTBs) decreased with increasing education level (none = 40 %; SHS = 11⋅5 %). PTBs showed slightly different trends by socio-demographic status. Older adolescents (16–19 years) recorded more PTBs (13⋅1 %) than adolescents in the younger bracket (5 %). Similarly, more PTB's were recorded among adolescents who were married (19⋅4 %) and employed (16⋅9 %) than among those who were single (13⋅8 %) and unemployed (10⋅9 %). LBWs were proportionally higher among adolescents who were younger than their older counterparts (22⋅2 % *v*. 14⋅8 %), married than single (19⋅4 % *v*. 13⋅8 %), employed than unemployed (16⋅9 % *v*. 15⋅6 %) and among those with high-income range than those without income (33⋅3 % *v*. 13⋅6 %).

**Table 1.** Differences in socio-demographic factors and birth outcomes (birth weight and gestational age)

Variables	Total	Birth weight		*P* value	Gestational age		*P* value
	*n* (%)	LBW	NBW		Preterm	Term	
Age				0⋅360^b^			0⋅487^b^
13–15 years	32 (7⋅7)	4 (22⋅2)	16 (77⋅8)		1 (5⋅0)	19 (95⋅0)	
16–19 years	384 (92⋅3)	37 (14⋅8)	213 (85⋅2)		37 (13⋅1)	246 (86⋅9)	
Marital Status				0⋅326^b^			0⋅428^b^
Single	316 (76⋅0)	28 (13⋅8)	175 (86⋅2)		26 (11⋅6)	199 (88⋅4)	
Married	100 (24⋅0)	13 (19⋅4)	54 (80⋅6)		12 (15⋅4)	66 (84⋅6)	
Occupation				0⋅257^b^			0⋅176^b^
Unemployed	298 (71⋅6)	33 (15⋅6)	163 (84⋅4)		24 (10⋅9)	196 (86⋅4)	
Employed	118 (28⋅4)	8 (16⋅9)	66 (83⋅1)		14 (16⋅9)	69 (83⋅1)	
Parity				0⋅845^b^			1⋅000^b^
1	316 (76⋅0)	30(14⋅9)	172(85⋅1)		29 (12⋅8)	198 (87⋅2)	
>1	100 (24⋅0)	11 (16⋅2)	57 (83⋅8)		9(11⋅8)	67 (88⋅2)	
Education				0⋅428^a^			**0⋅006**^a^
None	19 (4⋅6)	2 (16⋅7)	10 (83⋅3)		6 (40⋅0)	9 (60)	
Primary	56 (13⋅5)	3 (8⋅8)	31 (91⋅2)		2 (5⋅1)	37 (94⋅9)	
JHS	255 (61⋅3)	25 (14⋅5)	148 (85⋅5)		23 (12⋅2)	165 (87⋅8)	
SHS	86 (20⋅7)	11 (21⋅6)	40 (78⋅4)		7 (11⋅5)	54 (88⋅5)	
Income				0⋅517^a^			0⋅094^a^
No Income	310 (74⋅5)	28 (13⋅6)	178 (86⋅4)		23 (10⋅0)	206 (90⋅0)	
Below 100	41 (9⋅9)	4 (17⋅4)	19 (82⋅6)		7 (23⋅3)	26 (76⋅7)	
Between 100–500	61 (14⋅7)	8 (21⋅1)	30 (78⋅9)		8 (19⋅0)	34 (81⋅0)	
Between 500–1000	4 (1⋅0)	1 (33⋅3)	2 (66⋅7)		0	2 (100)	

HHS, Household Hunger Scale; LPI, Lived Poverty Index.

Bold values are significant at *P* < 0⋅05.

a *χ*^2^.

b Fischer's exact test.

### ANC service delivery and uptake, maternal factors and birth outcomes

Table 2 presents the frequencies of antenatal interventions, maternal factors and participants’ birth outcomes in the present study. Levels of participants who received nutrition education (*P* = 0⋅022), tetanus injection (*P* = 0⋅021), used insecticide-treated nets (*P* = 0⋅026) and mode of delivery (*P* = 0⋅009) varied significantly between rural and urban study participants. Generally, about half of pregnant adolescents (49⋅5 %) received nutrition education during antenatal services. Nearly 12 % more of pregnant adolescents from urban areas received nutrition education than their rural counterparts (*P* = 0⋅022). About 60⋅6 % received micronutrient supplements while only 46 % were taking their supplements daily. Four^(4)^ out of ten pregnant girls had not received tetanus injection. However, a more significant proportion of urban girls (*P* = 0⋅021) had received tetanus injection. About four to five out of ten had not received malaria tablets or insecticide-treated net (ITN). About 17 % of pregnant girls who had received the ITN were not using them. The use of ITN among rural girls was significantly higher (*P* = 0⋅026) than those in urban areas. Though not significant, LBW was higher among deliveries by rural girls (18⋅8 %) than girls (13⋅4 %). Also, preterm births proportion was higher among urban girls (13⋅1 %) than rural (11⋅9 %). About eight in ten pregnant girls delivered their babies using the vaginal method. Significantly, more rural girls (*P* = 0⋅009) than urban girls delivered through the vaginal method.

**Table 2.** Antenatal care interventions and uptake, maternal factors and birth outcomes between rural and urban-dwelling pregnant adolescents

Variable	Total *n* (%)	Rural *n* (%)	Urban *n* (%)	*P* value
Nutrition Education				**0⋅022**
Yes	206 (49⋅5)	74 (42⋅8)	132 (54⋅3)	
No	210 (50⋅5)	99 (57⋅2)	111 (45⋅7)	
Micronutrient Supplementation				0⋅186
Yes	252 (60⋅6)	98 (56⋅6)	154 (63⋅4)	
No	164 (39⋅4)	75 (43⋅4)	89 (36⋅6)	
Tetanus Injection				**0⋅021**
Yes	237 (57)	87 (50⋅3)	150 (61⋅7)	
No	179 (43)	86 (49⋅7)	93 (38⋅3)	
Insecticide-Treated Net				0⋅108
Yes	243 (58⋅4)	93 (53⋅8)	150 (61⋅7)	
No	173 (41⋅6)	80 (46⋅2)	93 (38⋅3)	
Malaria Tablet Intake				0⋅686
Yes	168 (40⋅4)	72 (41⋅6)	96 (39⋅5)	
No	248 (59⋅6)	101 (58⋅4)	147 (60⋅5)	
Daily Intake of Supplements				1
Yes	190 (45⋅7)	79 (45⋅7)	111 (45⋅7)	
No	226 (54⋅3)	94 (54⋅3)	132 (54⋅3)	
Use of ITN				**0⋅026**
Yes	172 (41⋅3)	83 (48⋅0)	89 (36⋅6)	
No	244 (58⋅7)	90 (52⋅0)	154 (63⋅4)	
Birth weight				0⋅495
LBW (<2⋅5 kg)	41 (15⋅2)	20 (17⋅1)	21 (13⋅7)	
Normal (≥2⋅5 kg)	229 (84⋅8)	97 (82⋅9)	132 (86⋅3)	
Gestational Age				0⋅862
Preterm (<37 weeks)	38 (12⋅5)	17 (11⋅9)	21 (13⋅1)	
Term (≥37 weeks)	265 (87⋅5)	126 (88⋅1)	139 (86⋅9)	
Maternal Morbidity				0⋅099
Yes	55 (17)	29 (21⋅3)	26 (13⋅9)	
No	268 (83)	107 (78⋅7)	161 (86⋅1)	
Delivery Method				**0⋅009**
Vaginal	285 (88⋅2)	142 (93⋅4)	143 (83⋅6)	
Caesarean	38 (11⋅8)	10 (6⋅6)	28 (16⋅4)	

LBW, Low Birth Weight.

Categorical data are presented as frequency (%).

Bold values are significant at *P* < 0⋅05.

### Impact of antenatal service delivery and uptake, and maternal factors and birth outcomes

Table 3 presents the effect of antenatal intervention uptake and maternal factors on birth outcomes. Adolescents who reported ill while pregnant showed a significantly larger proportion of LBWs (27⋅1 %) than those who did not (12⋅6 %; *P* = 0⋅024). Also, PTBs were over twice more among those reporting ill (24⋅1 %) than those who reported no illness (10 %; *P* = 0⋅011). LBW was significantly lower among participants who used ITNs (9⋅3 %; *P* = 0⋅038) as compared with those who did not (19 %). Also, LBW was slightly lower among adolescents who received nutrition education but higher among those who took micronutrient supplements, and malaria tablets, tetanus injection, than their counterparts who did not though these did not reach a statistical significance. Though not significant, participants who delivered through caesarean section (20⋅7 %) gave birth to a greater proportion of babies with LBW as compared with those who gave birth through the vaginal delivery method (15⋅8 %).

**Table 3.** Comparison of birth outcomes by antenatal care interventions uptake and maternal factors

Variables	Birth weight		*P* value	Gestational age		*P* value
	LBW	NBW		Preterm	Term	
Nutrition Education			0⋅499			1
Yes	19 (14)	117 (86)		19 (12⋅4)	134 (87⋅6)	
No	24 (17⋅9)	110 (82⋅1)		19 (12⋅7)	131 (87⋅3)	
Micronutrient Supplementation			0⋅29			0⋅375
Yes	29 (17⋅1)	141 (82⋅9)		26 (14⋅4)	158 (85⋅9)	
No	12 (12⋅0)	88 (88⋅0)		12 (10⋅1)	107 (89⋅9)	
Tetanus Injection			0⋅392			0⋅861
Yes	27 (16⋅9)	131 (83⋅1)		21 (12⋅1)	153 (87⋅9)	
No	16 (12⋅7)	96 (87⋅3)		17 (13⋅2)	112 (86⋅8)	
Insecticide-Treated Net			0⋅496			0⋅599
Yes	15 (13⋅3)	98 (86⋅7)		24 (13⋅6)	153 (86⋅4)	
No	26 (16⋅6)	131 (83⋅4)		14 (11⋅1)	112 (88⋅9)	
Malaria Tablet Intake			1			0⋅862
Yes	18 (15⋅4)	99 (84⋅6)		16 (12⋅9)	108 (87⋅1)	
No	23 (15⋅0)	130 (85⋅0)		22 (12⋅3)	157 (87⋅7)	
Daily intake of supplements			0⋅235			0⋅863
Yes	24 (18⋅2)	108 (81⋅8)		18 (12⋅9)	121 (87⋅1)	
No	17 (12⋅3)	121 (87⋅7)		20 (12⋅2)	144 (87⋅8)	
Use of ITN			**0⋅038**			0⋅061
Yes	10 (9⋅3)	96 (90⋅7)		14 (11⋅2)	111 (88⋅8)	
No	31 (19⋅0)	133 (81⋅0)		24 (13⋅5)	154 (86⋅5)	
Maternal morbidity			**0⋅024**			**0⋅011**
Yes	13 (27⋅1)	35 (72⋅9)		13 (24⋅1)	41 (75⋅9)	
No	28 (12⋅6)	194 (87⋅4)		25 (10⋅0)	224 (90⋅0)	
Mode of delivery			0⋅589			0⋅536
Vaginal	30 (15⋅8)	160 (84⋅2)		21 (9⋅9)	191 (90⋅1)	
Caesarean	6 (20⋅7)	23 (79⋅3)		4 (12⋅9)	27 (87⋅1)	

LBW, Low Birth Weight; NBW, Normal Birth Weight.

Bold values are significant at *P* < 0⋅05.

Table 4 shows the effect of the HHS, LPI, eating behaviour and MDD on birth outcomes. Among these variables, HHS had a statistically significant effect on birth weights (*P* = 0⋅006) and PTB (*P* = 0⋅028), whereby adolescent girls who suffered hunger had a larger proportion of LBW (no hunger = 12 %, moderate = 35⋅5 % and severe = 16⋅1 %; *P* = 0⋅006) and PTB (no hunger = 10 %, moderate = 24⋅4 % and severe = 13⋅2 %; *P* = 0⋅028). Likewise, poorer pregnant adolescent had significantly more LBW (*P* = 0⋅029) and PTB (*P* = 0⋅006). Although not statistically significant, LBW was lower among those with adequate MDD (11⋅4 %) as compared with those with inadequate MDD (17⋅9 %), while PTB was rather lower among those with inadequate MDD (11⋅6 %) than those whose MDD were adequate (13⋅7 %). Similarly, those who practiced PICA had more LBWs (16⋅2 %) and less PTBs (10⋅7 %) than those who did not (LBW = 14⋅6 %, *P* = 0⋅057; PTB = 13⋅6 %, *P* = 0⋅590).

**Table 4.** Comparison of birth outcomes by HHS, LPI, eating behaviour and dietary diversity

Variables	Birth Weight		*P* value	Gestational Age		*P* value
	LBW	NBW		Preterm	Term	
HHS			**0⋅003**^a^			**0⋅028**^a^
No Hunger	25 (12⋅0)	183 (88)		22 (10)	198 (90)	
Moderate Hunger	11 (35⋅5)	20 (64⋅5)		11 (24⋅4)	34 (75⋅6)	
Severe Hunger	5 (16⋅1)	26 (83⋅9)		5 (13⋅2)	33 (86⋅8)	
LPI			**0⋅029**^a^			**0⋅006**^a^
Low	15 (11⋅6)	114 (88⋅4)		13 (9⋅6)	123 (90⋅4)	
Moderate	5 (9⋅8)	46 (90⋅2)		3 (5⋅1)	56 (94⋅6)	
High	21 (23⋅3)	69 (76⋅7)		22 (20⋅4)	265(79⋅6)	
Food Aversion			0⋅995^b^			1⋅000^b^
Yes	19 (15⋅2)	106 (84⋅8)		17 (12⋅5)	119 (14⋅6)	
No	22 (15⋅2)	123 (84⋅8)		21 (12⋅6)	146 (87⋅4)	
Food Craving			0⋅818^b^			0⋅858^b^
Yes	27 (14⋅8)	155 (85⋅2)		25 (12⋅9)	169 (87⋅1)	
No	14 (15⋅9)	74 (84⋅1)		13 (11⋅9)	96 (88⋅1)	
Pica Practice			0⋅728^b^			0⋅590^b^
Yes	16 (16⋅2)	83 (83⋅8)		12 (10⋅7)	100 (89⋅3)	
No	25 (14⋅6)	146 (85⋅4)		26 (13⋅6)	165 (86⋅4)	
Minimum Dietary Diversity			0⋅170^b^			0⋅287^b^
Adequate	13 (11⋅4)	101 (88⋅6)		18 (13⋅7)	113 (86⋅3)	
Inadequate	28 (17⋅9)	128 (82⋅1)		20 (11⋅6)	152 (88⋅4)	

HHS, Household Hunger Scale; LPI, Lived Poverty Index; LBW, Low Birth Weight; NBW, Normal Birth Weight.

Bold values are significant at *P* < 0⋅05.

a *χ*^2^.

b Fischer's exact test.

### Predictors of adverse birth outcomes

Table 5 shows the findings of logistic regression on predictors of LBW. The use of ITN, LPI, HHS and maternal morbidity were significantly associated with delivering LBW babies. Pregnant adolescents who used ITN presented reduced odds (AOR 0⋅4; *P* = 0⋅013; 95 % CI 0⋅2, 0⋅9) of giving birth to babies with LBW compared with those who did not use them. Those who were poorer, using LPI presented increased odds (OR 2⋅5; *P* = 0⋅014; 95 % CI 1⋅1, 4⋅8) of delivering LBW babies than those with low LPI. Also, pregnant adolescents who were moderately hungry had a higher risk of delivering LBW babies (AOR 3⋅0; *P* = 0⋅028; 95 % CI 1⋅1, 8⋅1) compared with those who were not hungry. Maternal morbidity increased odds (AOR 2⋅7; *P* = 0⋅020; 95 % CI 1⋅2, 6⋅0) of babies with LBW as compared with those who did not experience any sickness.

**Table 5.** Predictors of low birth weight

Variable	Low birth weight (LBW)			
	Unadjusted OR (95 % CI)	*P* value	AOR (95 % CI)	*P* value
Use of Insecticide Net				
Yes	0⋅4(0⋅2–1⋅0)	**0⋅038**	0⋅4(0⋅2–0⋅8)	**0⋅013**
No	1		1	
Lived Poverty Index				
High	2⋅5(1⋅2–5⋅1)	**0⋅014**	1⋅9(0⋅8–4⋅6)	0⋅157
Moderate	1⋅0(0⋅3–2⋅7)	0⋅883	0⋅8(0⋅3–2⋅3)	0⋅657
Low	1		1	
Household Hunger Scale				
Moderate hunger	4⋅0(1⋅7–9⋅3)	**0⋅001**	3⋅0(1⋅1–8⋅1)	**0⋅028**
Severe hunger	1⋅4(0⋅5–4⋅0)	0⋅521	1⋅0(0⋅3–3⋅3)	0⋅971
No hunger	1		1	
Maternal Morbidity				
Yes	2⋅6(1⋅2–5⋅4)	**0⋅013**	2⋅7(1⋅2–6⋅0)	**0⋅020**
No	1		1	

Adjusted for Age and Community type, OR, Odds ratio; AOR, Adjusted odds ratio; CI, Confidence interval, statistically significant p values are bolded.

Table 6 presents a logistic regression analysis of the predictors of PTB. Maternal education status, LPI, HHS and maternal morbidity were significantly associated with delivering PTB. Pregnant adolescents with no formal education presented increased odds (AOR 9⋅0; *P* = 0⋅004; 95 % CI 2⋅1, 39⋅8) of having PTB compared with those with formal education. Adolescents who suffered hunger had increased odds (OR 2⋅9; *P* = 0⋅010; 95 % CI 1⋅3, 6⋅5) for PTB, compared with those who were not hungry. Poorer pregnant adolescents presented increased odds (OR 2⋅5; *P* = 0⋅014; 95 % CI 1⋅2, 5⋅3) of delivering preterm babies than those who were not poor. Experiencing maternal morbidity increased the odds (AOR 3⋅0; *P* = 0⋅011; 95 % CI 1⋅3, 7⋅0) of having PTB as compared with those who did not experience any form of morbidity.

**Table 6.** Predictors of preterm birth

Variable	Preterm birth (PTB)			
	Unadjusted OR (95 % CI)	*P* value	AOR (95 % CI)	*P* value
Education				
None	5⋅1(1⋅4–18⋅8)	**0⋅013**	9⋅0(2⋅1–39⋅8)	**0⋅004**
JHS	1⋅1(0⋅4–2⋅6)	0⋅874	1⋅3(0⋅5–3⋅2)	0⋅646
Primary	0⋅4(0⋅1–2⋅1)	0⋅292	0⋅4(0⋅1–2⋅4)	0⋅325
SHS	1		1	
Lived Poverty Index				
High	2⋅5(1⋅2–5⋅3)	**0⋅014**	2⋅3(0⋅9–5⋅8)	0⋅087
Moderate	0⋅4(0⋅2–2⋅0)	0⋅370	0⋅4(0⋅1–1⋅5)	0⋅182
Low	1		1	
Household Hunger Scale				
Moderate hunger	2⋅9(1⋅3–6⋅5)	**0⋅01**	2⋅3(0⋅9–6⋅2)	0⋅092
Severe hunger	1⋅4(0⋅5–3⋅8)	0⋅558	0⋅8(0⋅2–2⋅9)	0⋅772
No hunger	1		1	
Maternal Morbidity				
Yes	2⋅8(1⋅3–6⋅0)	**0⋅006**	3⋅0(1⋅3–7⋅0)	**0⋅011**
No	1		1	

Adjusted for Age and Community type, OR, Odds ratio; AOR, Adjusted odds ratio; CI, Confidence interval, statistically significant p values are bolded.

## Discussion

The present study identified predictors of adverse birth outcomes among pregnant adolescents in Ghana. The findings indicated that the use of ITN, maternal education status, hunger (HHS), poverty (LPI) and maternal morbidity were the main determinants of adverse birth outcomes (LBW and PTB) among pregnant adolescents in Ghana. The prevalence of LBW and PTB reported in the present study was 15⋅2 and 12⋅5 %, respectively. Similarly, the LBW prevalence for Western Africa and Ghana reported in 2019 were 15⋅2 and 14⋅2 %, respectively^(49)^. Our population of adolescents is, therefore, similar to the national average with regards to LBW and lower for PTB. Compared with other studies around the world, our findings regarding LBW and PTB were higher^(32,50)^ but lower from other results^(51,52)^. Within Ghana, a study in Cape Coast reported a similar LBW prevalence of 14⋅3 % among pregnant adolescents^(36)^.

Generally, access to ANC interventions among pregnant adolescents used in the study was relatively low. For example, less than half (49⋅5 %) of the participants reported having received nutrition education at ANC. The uptake of the interventions by adolescents was also low. Four in ten participants did not take their micronutrient supplements, nor used the ITNs, and a few took their malaria tablets (40⋅4 %). The low compliance may negate the benefits of attending ANC, as the mere attendance to ANC sessions is not the fundamental objective. This calls for programmes, including pregnant women's education in general, on the benefits of complying with these interventions. The low compliance may also be due to the fewer number of ANC sessions attended. Pregnant adolescents are likely to delay pregnancy disclosure due to uncertainty and vulnerability, especially during the first trimester, lack of funds and stigmatisation^(30)^. Compared with their adult counterparts, pregnant adolescents are likely to access ANC services later during pregnancy^(53,54)^. Among all the ANC interventions and compliance assessed, adolescent girls from the urban areas were better off than their rural counterparts. More urban pregnant adolescents received nutrition education (0⋅022), tetanus injection (0⋅021) and had access to ITNs (although more rural girls actually used the ITNs) compared with their rural folks. Tanya and Goldenberg^(17)^ identified the inability to pay for services, lack of transport and prior negative experiences as barriers to healthcare services among rural women. For the present study, we think low socioeconomic status and less access to health care in rural areas are possible reasons for the lower uptake of interventions by rural girls.

The consistent and appropriate use of ITN has been one of the efficient means in preventing malaria infection by limiting the contact and bite of mosquitoes^(55)^. Since 2000, mass and continuous distribution networks have substantially increased access to ITNs in Ghana^(56)^. Notwithstanding these achievements, a significant gap exists between ITN access and usage. In our study, 58 % of the pregnant adolescents reported having received ITN, while 41 % were using them. Low use of ITN has been reported and associated with discomfort from the smell of chemical used, heat and difficulty installing the net^(57)^. Younger women (under 19 years of age) and women with less education or less hurdles to ITN usage include low awareness of ITNs by women, household or cultural constraints such as low social status or economic dependence, age, marital status, education, employment status, knowledge of malaria/ITN and IPTp reception^(58)^. However, our study reported a 10 % less LBW deliveries in ITN non-users than among ITN users (9⋅3 % *v*. 19 %; *P* = 0⋅037). This disparity may be related to the possibility that despite these adolescents receiving antenatal interventions, they were at a higher risk of other relating factors such as maternal underweight, hunger, poverty directly associated with LBW compared with those who did not receive antenatal interventions, and these factors may have confounding effects on their birth outcomes rather than the antenatal interventions.

*Plasmodium falciparum* infection during pregnancy is a prominent cause of LBW. In our findings, the use of ITN was significantly associated with LBW; those who used the ITN presented reduced odds of delivering babies with LBW. Similar to this result, other studies^(59–61)^ have reported the association of ITN use with birth outcomes. For instance, a significant reduction in the risk of LBW in healthy malaria transmission regions has been associated with the use of antimalarial intermittent pregnancy prevention (IPTp) and ITN during pregnancy^(59)^. A higher reduction rate of LBW (23 %) and fetal loss (33 %) with the use of ITN has also been reported among women of low gravidity in Africa^(60)^. Kubi Appiah and others also reported increased odds of normal birth weight deliveries (AOR 2⋅17; 95 % CI 0⋅03, 0⋅92) to be associated with women who received a long-lasting ITN from ANC clinics^(61)^.

The study predicted that among all socio-demographic factors used, the education status of the mother was significantly associated with PTB. Among pregnant adolescents who had no formal education, 40 % delivered PTB babies. This suggests that maternal education level affects birth outcomes. Access to education can expose an individual to information healthier lifestyles during pregnancy, preventing adverse effects. Similar results of low maternal education level associated with adverse birth outcomes were reported in Quebec, Canada^(62)^, and in two meta-analysis^(63,64)^. We understand the correlation between maternal education and maternofetal outcomes by the more likely disposition of women of higher education having a greater understanding of prenatal care, early initiation of prenatal care, more ANC consultations as prescribed and higher socioeconomic status^(64,65)^. Contrary to our findings, maternal education among Mexican American had little effect on birth outcomes^(66)^. They observed that the Mexican American presented the least infant mortality rate as compared with the other populations used^(66)^.

Hunger and poverty presented significant associations with adverse birth outcomes. Hunger led to an increased likelihood of having adverse birth outcomes in the present study. Pregnant adolescents who experienced hunger and were poorer using HHS and LPI, respectively, presented significantly higher odds of giving birth to LBW and preterm babies than those who did not experience any hunger or deprivation. The possible reason for more adverse birth outcomes among pregnant adolescents who experienced poverty and hunger is inadequate maternal dietary intake and low nutritional quality diet, as these have been related to adverse birth outcomes^(11)^. During pregnancy, the intake of adequate dietary diversity diet offers the required micronutrients that are important for the growth and development of the fetus. However, our findings did not present a significant relationship between dietary diversity and adverse birth outcomes though a greater proportion of LBW babies were found among adolescents with inadequate dietary diversity as compared with those with adequate dietary diversity. Contrary to our findings, Quansah and Boateng^(67)^ found inadequate dietary diversity to be associated with higher odds of LBW babies. Maternal nutritional status can have an impact on fetal development. The proper growth and development of the fetus requires the consumption of food items from the various food groups in order to prevent deficiencies of essential micronutrients. Thus, adequate dietary intake is critical in preventing adverse birth outcomes. In developing countries, poor nutritional status is a determining factor of LBW due to low nutrient flow to the developing fetus^(68)^. Experiencing hunger during pregnancy can be devastating and can be subsequently linked to poor nutritional status as the pregnancy period requires a significant increase in macronutrients and adequate micronutrients. According to a study on fetal response to satiation and hunger, 74 % of pregnant women experienced an increase in fetal activity when they were hungry. The paper further stated that women with increased fetal activity gave birth to smaller babies^(69)^. Studies have related poverty to adverse birth outcomes such as LBW, PTB and fetal loss^(17,70)^. Tanya^(17)^ further asserted that the poor might have less access to healthcare systems and maternal care during emergencies. A high risk of malnutrition and subsequent adverse effect on babies leading to nutritional and developmental deficiencies have also been related to poverty^(71)^.

Maternal morbidity was significantly associated with birth outcomes in the present study. The odds of having LBW and PTB among those who got ill during pregnancy were thrice more than those who did not experience any illness. This implies that the health of the mother can be a contributory factor to birth outcomes. According to Tinker^(20)^, maternal malnourishment and insufficient maternal care are high risks for the development of diseases and death during delivery. This is expected since maternal illness can affect fetal growth and development. Some of the conditions that these pregnant adolescents went through during pregnancy were pregnancy induced hypertension, antepartum haemorrhage and prolonged labour. These findings seem to be consistent with earlier research which reported that participants who experienced morbidity had about five to seven times increased risk of adverse birth outcomes^(21,72,73)^.

Generally, the effect of geographical location, environmental conditions, socio-cultural practices, eating behaviour and maternal factors on birth outcomes have been mixed. For instance, in the present study, LBW was more prevalent among rural girls (17 %) than the urban participants (13⋅7 %), similar to a study conducted in Malaysia^(74)^, but contrary to other studies in Ghana^(36,75,76)^, which reported a higher prevalence of LBW among urban than rural participants. A higher prevalence of LBW is expected in rural areas due to poorer socioeconomic conditions, food insecurity, low nutrition knowledge and limited access to health care^(77–79)^. In the present study, the prevalence of caesarean delivery, 11⋅8 %, was similar to studies conducted in Ghana^(76)^ but lower than two studies conducted in Bangladesh^(80)^ and Pakistan^(70)^, which recorded 23⋅9 and 26⋅4 %, respectively. A greater proportion of caesarean deliveries in this study (16⋅4 %) were from the urban centres. This may seem to be due to urbanisation^(81)^ and access to qualified personnel to carry out the section. Hasan^(80)^ also presented similar findings where the rate of caesarean deliveries was relatively high in urban women than in rural women. Caesarean sections are less accessible among poor or low-income countries^(82)^. Generally, about 21 % of those who delivered through caesarean section gave birth to LBW babies. Literature suggests that caesarean sections are usually performed as emergencies without adequate preparations and can lead to a challenge in the newborn's proper initial development^(83)^.

The overall nutritional status such as weight gain, health, parity, previous birth outcomes and biochemical status of the mother during pregnancy may affect birth outcomes. Some studies have presented an increased odds of obstetric, placental and medical complications with increasing parity^(84–86)^. Furthermore, poor socioeconomic status and its consequences, such as less access to health facilities and low levels of education have been associated with adverse birth outcomes^(87,88)^. Moreover low birth weight have been associated with pregnant women with chronic anaemia, diabetes and hypertension^(84,89)^.

In summary, this study explored the predictors of adverse birth outcomes among pregnant adolescents, and among many factors found that hunger, poverty and lack of formal education predicted preterm births. Pregnant girls with no formal education had nine times increased odds for PTB compared with those with at least secondary education. Girls with high LPI had two and a half increased odds for PTB compared with those with no poverty, and those who experienced moderate hunger were about two and a half times increased odds for PTB compared with those with no hunger. Finally, those who were ill during pregnancy were thrice increased odds for PTB compared those who were not ill. For LBW, the significant predictors were similarly hunger, poverty and maternal morbity. The levels of the odds recorded for the predictors of LBW were simlar to that of the PTB. On the other hand, the use of ITN reduced the odds for LBW deliveries by about 60%.

## Limitation

The study had a significant dropout as less than 75 % of baseline participants were followed up for the birth outcomes study. Future studies should be designed to reduce this, either by increasing the sample size or improve measures for tracking participants.

## Conclusion

The risk of adverse birth outcomes observed among the pregnant adolescents were associated with ANC compliance and maternal socioeconomic factors. Strengthening antenatal services, promoting girl education, livelihood support for pregnant girls, and interventions to improve socio-economic status and to address hunger and poverty are broad and targeted interventions to both reduce the incidence of adolescent pregnancies, and reduce the risk for adverse birth outcomes among those who still become pregnant. Focused care to encourage attendance to ANC and improve compliance to ANC interventions by pregnant adolescents is strongly recommended. Food security and hunger should be evaluated during antenatal services to identify and address the vulnerable.
